# The Dynamic Mutation Characteristics of Thermonuclear Reaction in Tokamak

**DOI:** 10.1155/2014/841891

**Published:** 2014-04-29

**Authors:** Jing Li, Tingting Quan, Wei Zhang, Wei Deng

**Affiliations:** ^1^College of Applied Sciences, Beijing University of Technology, Beijing 100124, China; ^2^College of Mechanical Engineering, Beijing University of Technology, Beijing 100124, China; ^3^Beijing Electrical Research Institute, Beijing 100124, China

## Abstract

The stability and bifurcations of multiple limit cycles for the physical model of thermonuclear reaction in Tokamak are investigated in this paper. The one-dimensional Ginzburg-Landau type perturbed diffusion equations for the density of the plasma and the radial electric field near the plasma edge in Tokamak are established. First, the equations are transformed to the average equations with the method of multiple scales and the average equations turn to be a *Z*_2_-symmetric perturbed polynomial Hamiltonian system of degree 5. Then, with the bifurcations theory and method of detection function, the qualitative behavior of the unperturbed system and the number of the limit cycles of the perturbed system for certain groups of parameter are analyzed. At last, the stability of the limit cycles is studied and the physical meaning of Tokamak equations under these parameter groups is given.

## 1. Introduction

Periodic solution theory is mainly about the existence and stability of periodic solution of dynamical systems. The bifurcation theory of periodic solution, as the main method to study the periodic solution, reveals the connection between the topology of the solutions and the parameters. The investigation and application of bifurcations and chaos of nonlinear dynamical systems are frontier topics in the world.

Recently, a large number of important results of multiple limit cycles of polynomial planar vector fields have been achieved. Arnol'd [1] examined the problem on equivariant fields and their topologically versal deformations in the functional space of all equivariant fields and these results yield the first approximation for the stability loss problem. Perko [2] studied the local bifurcation and global behavior of one-parameter families of limit cycles of a planar analytic system. They obtained some new results on the global behavior of one-parameter families of limit cycles. Wang and Mao [3] gave an algorithm for computing the Lyapunov values and a new criterion for determining the centers of planar polynomial systems which have quartic nonlinear terms. They found that there are 11 small limit cycles in a kind of planar polynomial systems. Chan et al. [4] showed that the numerical examples of different quadratic differential systems had three limit cycles surrounding one singular point. Li [5] investigated Hilbert's 16th problem and bifurcations of planar polynomial vector fields and gave the detection function method of two dimensional Hamiltonian systems. Armengol and Joan [6] perturb a vector field with a general polynomial perturbation of degree *n* and study the maximum number of limit cycles that can bifurcate from the period annulus of the origin in terms of *k* and *n*. Their approach is based on the explicit computation of the Abelian integral that controls the bifurcation and on a new result for bounding the number of zeroes of a certain family of real functions. Han and Li [7] obtained some new lower bounds of Hilbert number *H*(*m*), and *H*(*m*) grows at least as rapidly as (1/2ln⁡2) (*m* + 2)^2^ln⁡(*m* + 2) for all large *m*. Zhao and Fan [8] studied the number of small amplitude limit cycles in arbitrary polynomial systems with degree *m*, denoted by *M*(*m*), and obtained the lower bounds for *M*(6) − *M*(14) and proved that *M*(*m*) ≥ *m* ^2^ if *m* ≥ 23. They also showed that the least growth order of *M*(*m*) is equal to that of *H*(*m*) (in [9]); however, the growth coefficient of *M*(*m*) is 25/18 that of *H*(*m*).

The symmetry of dynamical systems is also widely studied by many researchers and a lot of results have been achieved. Li et al. [10] analyzed a *Z*_6_-equivariant perturbed polynomial Hamiltonian system of degree 5 and found that there exist at most 24 limit cycles. Li et al. [9] investigated a rotor-active magnetic bearings (AMB) system with the time-varying stiffness and found that there exist, respectively, at least 17, 19, 21, and 22 limit cycles under four groups of parametric controlling conditions. Li et al. [11] investigated the bifurcation of multiple limit cycles of a *Z*_2_-equivariant perturbed polynomial Hamiltonian system of degree 5 and found that the *Z*_2_-equivariant fields have up to 23 limit cycles. Li et al. [12] studied bifurcations of limit cycles at three fine focuses for a class of *Z*_2_-equivariant nonanalytic cubic planar differential systems proved that there exist 12 small amplitude limit cycles created from the critical points.

In recent years, theories are widely used in mechanical systems. An important issue is the bifurcation of multiple limit cycles for Tokamak system. The Tokamak is the most promising device so far to attain the conditions for fusion. It is a toroidal device (shaped like a car tire) in which a vacuum vessel contains a plasma ring confined by twisting magnetic fields. In the past 20 years, researches and applications of the Tokamak have great achievements, especially with the implementation of the construction named EAST (Experimental Advanced Superconducting Tokamak). EAST is one of Chinese national fusion projects.

In this paper, the mechanism of the transition from L-mode to H-mode in Tokamak is an important and difficult problem. We mainly discussed the transition phenomenon between low confinement mode (L-mode) and high confinement mode (H-mode) observed in Tokamak. S. Itoh and K. Itoh [13] presented a new model of the transition from L-mode to H-mode in the Tokamak plasmas and discussed the catastrophic phenomenon of it. Viana [14] used a Hamiltonian description for magnetic field lines in a large aspect ratio Tokamak for describing the effect of resonant helical windings in a perturbative way, taking into account the toroidal correction. Zhang and Cao [15] found that in Tokamak there existed not only the static L-mode to H-mode transition but also to the dynamic L-mode to H-mode transition, and the Hopf bifurcation and limit cycle oscillations correspond to the L-mode to H-mode transition near the plasma edge in Tokamak.

This paper focuses on the bifurcations of multiple limit cycles for a Ginzburg-Landau type perturbed transport equation which can describe the L-mode to H-mode transition near the plasma edge in Tokamak. The average equation of Tokamak system turns out to be a *Z*_2_-equivariant perturbed Hamiltonian system. Using the bifurcation theory and the method of detection function, the number of limit cycles of the average equation under a certain group of parameters is given. The stability of these limit cycles is analyzed and the diffusion coefficients of H-mode and L-mode are obtained.

## 2. Equation of Motion and Perturbation Analysis

We get the nondimensional formulations by using the method in [16]. The one-dimensional Ginzburg-Landau type perturbed diffusion equations for the density of the plasma and the radial electric field near the plasma edge in Tokamak can be written as

(1) $$\begin{array}{c} \frac{\partial n}{\partial t}=\frac{\partial }{\partial x}\left[D\left(E_{r}\right)\frac{\partial n}{\partial x}\right]+f_{1}\cos\Omega_{1}t,\\ \gamma \frac{\partial E_{r}}{\partial t}=-N\left(E_{r},g\right)+\mu_{1}\frac{\partial^{2}E_{r}}{\partial t^{2}}+f_{2}\cos\Omega_{2}t, \end{array}$$

where *n* and *E*_*r*_ are the density of the particle near the plasma edge and the normalized radial electric field, respectively. *D*(*E*_*r*_) and *μ*_1_ are the diffusion coefficients of the density and electric field, *N*(*E*_*r*_, *g*) is the total current effect, and *f*_1_, *Ω*_1_, *f*_2_, and *Ω*_2_ are the amplitudes and frequencies of the particle perturbation and the controlling radial electric field. It is known that *D*(*E*_*r*_), *N*(*E*_*r*_, *g*), and *γ*,respectively, satisfy the following equations:

(2) $$\begin{array}{c} D\left(E_{r}\right)=\left(\frac{D_{\max}+D_{\min}}{2}+\frac{D_{\max}-D_{\min}}{2}\right)\tan D\left(E_{r}\right),\\ N\left(E_{r},g\right)=g-g_{0}+\left(\beta E_{r}^{3}-\alpha E_{r}\right),\\ g\left(n\right)=\frac{3}{n^{2}}\frac{\partial n}{\partial x},\;\;\gamma =\left(1+\frac{v_{A}^{2}}{c^{2}}\right)\frac{B_{P}^{2}}{B^{2}}, \end{array}$$

where *D*_max⁡_ and *D*_min⁡_, respectively, denote the diffusion coefficients of H-mode and L-mode, the parameters *v*_*A*_, *c*, *B*_*P*_, *B*, *α*, *β*, and *g*_0_ are constants, and *v*_*A*_, *c*, *B*_*P*_, and *B* are the Alfven velocity, the light velocity, the magnetic field which is parallel to the poloidal direction in Tokamak, and the characteristic magnetic field, respectively.

In order to analyze the diffusion of the particle and the stability and bifurcations of the normalized radial electric field near the plasma edge in Tokamak, some transformations may be introduced as follows:

(3) $$\begin{array}{c} n=\frac{1}{V},\;\;E_{r}=U,\\ V=V_{0}+A\left(x\right)v\left(t\right),\;\;U=G\left(x\right)u\left(t\right), \end{array}$$

where *A*(*x*) *v*(*t*) and *G*(*x*) *u*(*t*) are small perturbed terms. Substituting (2) and (3) into (1), we have the following equations:

(4a) v˙=a1u+a2uv+a3v+a4v2+a5v3+a6uv2+a7vcos⁡Ω1t+a8v2cos⁡Ω1t+a9cos⁡Ω1t+a0

(4b) u˙=b1v+b2u+b3u3+b4cos⁡Ω2t+b0,

Eliminate *v* and $\dot{v}$; then we can get

(5) u¨−ε(μ+β2u˙+β3u+β4uu˙+β5u2+β6u˙2)u˙+ω2u   −εα2u2−εα3u3+ε(δ1cos⁡Ω1t+δ2cos⁡Ω2t)u  =εF1cos⁡Ω1t+εF2cos⁡(Ω2t+φ2)+ε2α0.

We assume that the uniform solution of (5) can be represented in the form

(6) x(t,ε)=y0(T0,T1,T2,…)+εx1(T0,T1,T2,…)+ε2x2(T0,T1,T2,…)+⋯,

where *T*_*i*_ = *ε* ^*i*^ *t*, *i* = 0, 1, 2,… .

Then, the differential operators are given as

(7) ddt=∂∂T0∂T0∂t+∂∂T1∂T1∂t+∂∂T2∂T2∂t+⋯=D0+εD1+ε2D2+⋯,d2dt2=(D0+εD1+ε2D2⋯)2=D02+2εD0D1+ε2(D12+2D0D2)+⋯,

where *D*_*k*_ = ∂/∂*T*_*k*_, *k* = 0, 1.

The main work of this paper focuses on the 1/2 subharmonic resonance-primary parametric resonance, because this resonant case is the most common case which may be exhibited in system (5). To simplify the procedure of the analysis, without loss of generality, we may assume that

(8) $$\begin{array}{c} \omega =1+\varepsilon \sigma ,\;\;\Omega_{1}=\Omega_{2}=2, \end{array}$$

where *σ* is a detuning parameter. Substituting (6)–(8) into (5), and we get the averaged equations of (5):

(9a) x˙=(j+j~)x+(k+k~)y+(l+l~)x3+(m+m~)x2y+(n+n~)xy2+(p+p~)y3+(q+q~)x(x2+y2)2+4q~(xy4−3x3y2)+(r+r~)y(x2+y2)2+4r(x4y−3x2y3),

(9b) y˙=(j−j~)y+(k−k~)x+(l−l~)y3+(m−m~)xy2+(n−n~)x2y+(p−p~)x3+(q−q~)y(x2+y2)2−4q~(x4y−3x2y3)+(r−r~)x(x2+y2)2+4r(xy4−3x3y2).

Then (9a) and (9b) can be rewritten as follows:

(10a) x˙a01y+a03y3+a05y5 −εx(−a50x4−a41x3y−a32x2y2−a23xy3    −a14y4−a30x2−a21xy−a12y2−a10),

(10b) y˙=b10x+b30x3+b50x5−εy(−b05y4−b14xy3−b23x2y2−b32x3y   −b41x4−b03y2−b12xy−b21x2−b01).

Let *a*_41_ = *a*_23_ = *a*_21_ = *b*_14_ = *b*_32_ = *b*_12_ = 0 and *a*_10_ = *b*_01_; the system ((10a) and (10b)) turns out to be a *Z*_2_-equivariant perturbed Hamiltonian system with 17 free parameters.

## 3. The Dynamic Characteristics of Thermonuclear Reaction in Tokamak

In Section 2, the Ginzburg-Landau Tokamak system turns out to be a *Z*_2_-equivariant Hamiltonian system of degree 5. We will give a procedure of controlling parameters to obtain more limit cycles of the system ((10a) and (10b)).

### 3.1. The Method of Detection Functions

In this section, the method of detection functions will be described briefly based on references [8, 17]. Let *H*(*x*, *y*) be a real polynomial of degree *n*, and let *P*(*x*, *y*) and *Q*(*x*, *y*) be two different real polynomials of degree *m*, respectively. We consider a perturbed Hamiltonian system in the following form:

(11a) dxdt=∂H∂y+εP(x,y,λ),

(11b) dydt=−∂H∂x+εQ(x,y,λ),

where 0 < *ε* ≪ 1 is a small parameter and the level energy curves *H*(*x*, *y*) = *h* of the unperturbed Hamiltonian system ((10a) and (10b))_*ε*=0_ contain at least a family of closed orbits Γ_*h*_ for *h* ∈ (*h*_*l*_, *h*_*r*_).

Consider the Abelian integral

(12) I(h)=∫ΓhP(x,y)dy−Q(x,y)dx=∬H≤h(∂P(x,y)∂x+∂Q(x,y)∂y)dx dy.

We define the function

(13) $$\begin{array}{c} \lambda =\lambda \left(h\right)=\frac{I\left(h\right)}{\iint_{D^{h}}dx\;dy}, \end{array}$$

which is called a detection function corresponding to the periodic family {Γ^*h*^}. The graph of *λ* = *λ*(*h*) in the plane (*h*, *λ*) is called a detection curve, where *D* ^*h*^ is the area inside Γ^*h*^.

**Theorem 1 (bifurcation of limit cycles)**

We have the following three statements on the local and global bifurcations.

1. If *I*(h*) = 0 and *I*′(h*) ≠ 0, then there exists a limit cycle *L*_h*_ of system ((11a) and (11b)) such that *L*_h*_ → Γ_h*_ as *ε* → 0. Conversely, if there exists a limit cycle *L*_h*_ of system ((11a) and (11b)) such that *L*_h*_ → Γ_h*_ as *ε* → 0, then *I*(h*) = 0, where h* ∈ (*h*_*l*_, *h*_*r*_).
2. If *I*(h*) = *I*′(h*) = *I*′′(h*) = ⋯ = *I* ^(*k*−1)^(h*) = 0 and *I* ^(*k*)^(h*) ≠ 0, then, for *ε* sufficiently small, system ((11a) and (11b)) has at most *k* limit cycles in the neighborhood of Γ_h*_.
3. The total number of isolated zeros of the Abelian integral is an upper bound for the number of limit cycles of system ((11a) and (11b)) after taking into account their multiplicity.

### 3.2. The Qualitative Behavior of Unperturbed System

We consider the unperturbed system of ((10a) and (10b)) as

(14a) x˙=a01y+a03y3+a05y5,

(14b) y˙=b10x+b30x3+b50x5,

where *a*_01_, *a*_03_, and *a*_05_ satisfy *a*_03_^2^ > 4*a*_01_ *a*_05_, *a*_03_ *a*_05_ < 0, and *a*_01_ *a*_05_ > 0 and *b*_10_, *b*_30_, and *b*_50_ satisfy *b*_30_^2^ > 4*b*_10_ *b*_50_, *b*_30_ *b*_50_ < 0, and *b*_10_ *b*_50_ > 0.

Equation ((14a) and (14b)) is a Hamiltonian system with Hamiltonian function

(15) H(x,y)=12(a01y2−b10x2)+14(a03y4−b30x4)+16(a05y6−b50x6).

Let

(16) $$\begin{array}{c} x_{1}=\sqrt{\frac{-b_{30}+\sqrt{b_{30}^{2}-4b_{10}b_{50}}}{2b_{50}}},\\ x_{2}=\sqrt{\frac{-b_{30}-\sqrt{b_{30}^{2}-4b_{10}b_{50}}}{2b_{50}}},\\ y_{1}=\sqrt{\frac{-a_{03}+\sqrt{a_{03}^{2}-4a_{01}a_{05}}}{2a_{05}}},\\ y_{2}=\sqrt{\frac{-a_{03}-\sqrt{a_{03}^{2}-4a_{01}a_{05}}}{2a_{05}}}. \end{array}$$

It is easily seen that there exist 25 singular points of system (15). Based on the analysis of the stability, it is found that 13 singular points, namely, (0, 0), (*x*_1_, 0), (±*x*_2_, *y*_1_), (0, *y*_2_), and (±*x*_1_, *y*_2_), and their *Z*_2_-symmetric points are the centers and 12 singular points, namely, (*x*_2_, 0), (0, *y*_1_), (±*x*_1_, *y*_1_), and (±*x*_2_, *y*_2_), and their *Z*_2_-symmetric points are the saddle points.

In this paper, we only consider a special case that

(17) UP=(a01,a03,a05,b10,b30,b50)=(−1.01,5.49,−3.76,3.01,−8.01,3.75).

**Proposition 2**

**Figure 1:**
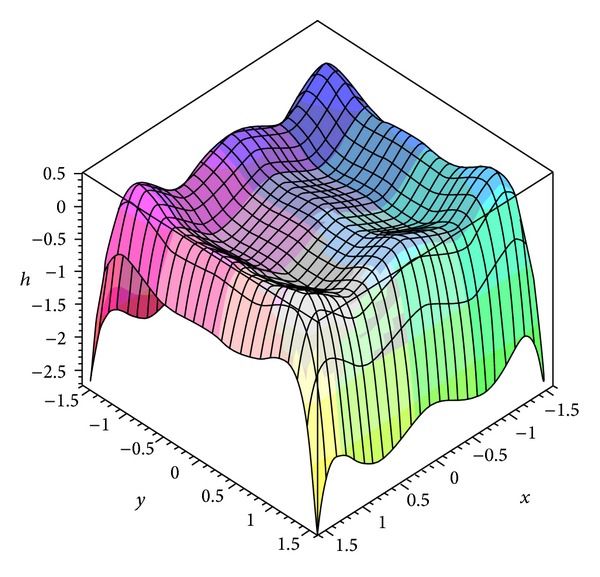
The diagram of the Hamiltonian function (15) is given.

**Figure 2:**
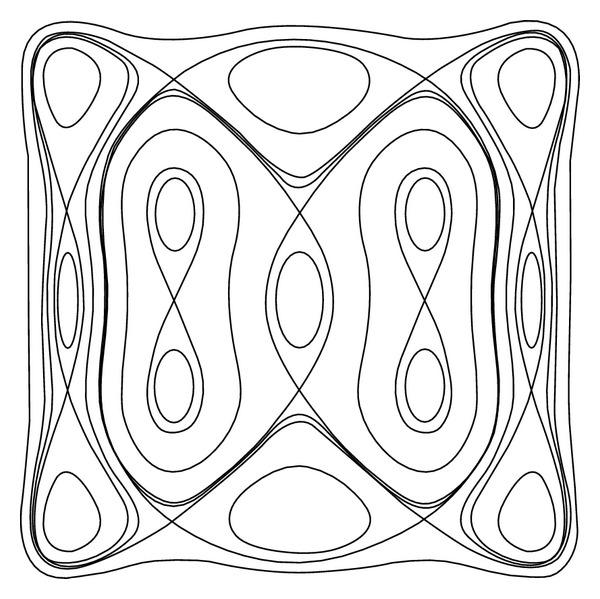
Families of closed orbits defined by system ((10a) and (10b)) with* UP *is given.

Under the conditions of* UP*, the Hamiltonian function (15) has the diagram as shown in Figure 1 and the *Z*_2_-equivariant Hamiltonian vector field ((14a) and (14b)) has the phase portraits as shown in Figure 2.

**Figure 3:**
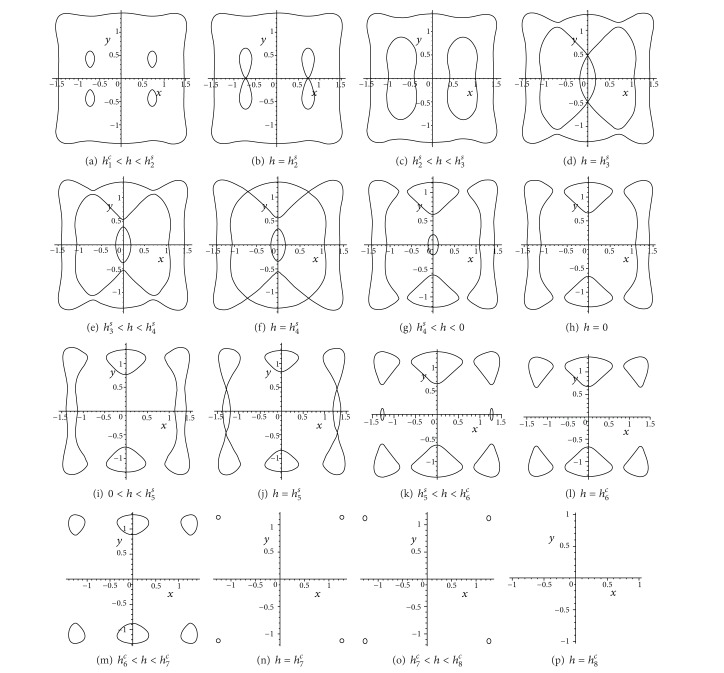
Different schemes of ovals defined by ((11a) and (11b)) as *h* varied with* UP *are given.

Figure 3 illustrates the changing process of the phase portraits for system ((14a) and (14b)) as the variable *h* changes from −*∞* to +*∞*. In the aforementioned case, there are nine different families (Γ_*j*_^*h*^) (*j* = 0, 1,…, 8) of closed orbits and several homoclinic or heteroclinic loops for unperturbed system ((14a) and (14b)) as the variable *h* changes from −*∞* to +*∞*.

Notice that as *h* increases, the periodic orbits Γ_1*i*_^*h*^, Γ_2*i*_^*h*^, and Γ_4_^*h*^ expand outwards and all other periodic orbits contract inwards.

### 3.3. The Qualitative Behavior of Perturbed System ((10a) and (10b))

Based on the results obtained above, we can analyze the qualitative nonlinear characteristics of the perturbed system ((10a) and (10b)). The detection functions corresponding to the aforementioned nine types of period families {Γ_0_^*h*^} − {Γ_8_^*h*^} are obtained as follows:

(18) $$\begin{array}{c} \lambda_{i}=\lambda \left(h\right)=\frac{\iint_{D_{i}^{h}}F\left(x,y\right)dxdy}{\iint_{D_{i}^{h}}dx\;dy}=\frac{\varphi_{i}\left(h\right)}{\phi_{i}\left(h\right)},\;\left(i=0,\ldots ,8\right), \end{array}$$

where *F*(*x*, *y*) = *P*(*x*, *y*)*dx* + *Q*(*x*, *y*)*dy* and *D* ^*h*^ is the area inside Γ^*h*^.

Denote that

(19) PG=(c40,c04,c22,c20,c02)=(76.05823,−48.67855,25.95000,−156.46000,15.15662).

**Figure 4:**
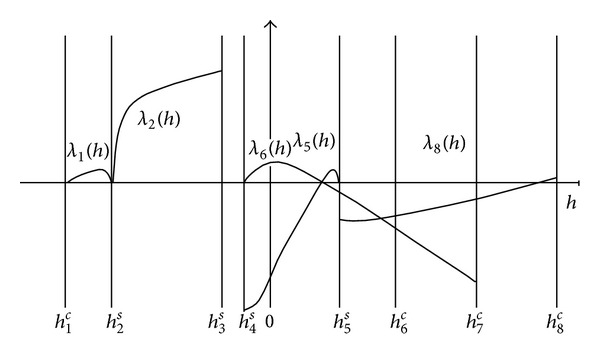
Graphs of detection curves of system ((9a) and (9b)) with parameter conditions* UP* and* PG *are given.

It follows that, under the parameter conditions of* UP *and* PG*, the system ((10a) and (10b)) has the graphs of detection curves as shown in Figure 4.

We can see from Figure 4 that when

(20) λ~∈(−54.39948,min⁡(max⁡λ1(h),max⁡λ5(h),   max⁡λ6(h),λ7(h5s))),

in the (*h* − *λ*)-plane, the straight line $\lambda =\tilde{\lambda }$ intersects the curves *λ* = *λ*_1_(*h*), *λ* = *λ*_5_(*h*), and *λ* = *λ*_6_(*h*) at two points and the curves *λ* = *λ*_2_(*h*) and *λ* = *λ*_8_(*h*) at one point, respectively. With the *Z*_2_-equivariance of ((10a) and (10b)) and from the results above, we have the following conclusion.

**Proposition 3**

**Figure 5:**
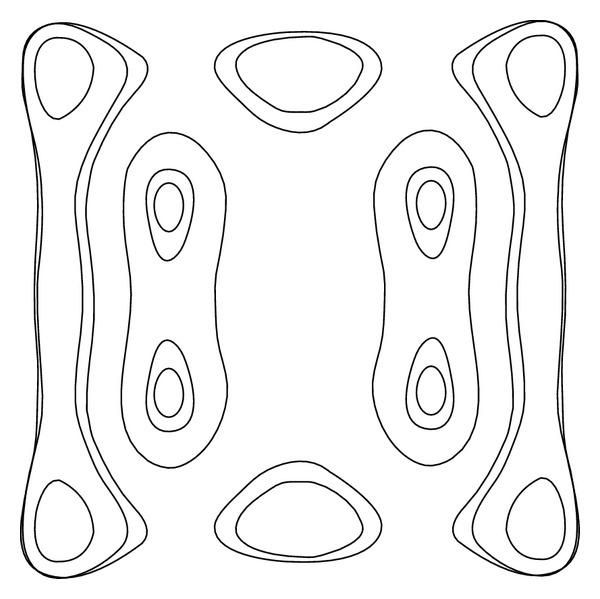
Configuration of 22 limit cycles of system ((10a) and (10b)) with parameter conditions* UP* and* PG* is given.

When $\lambda =\tilde{\lambda }$ satisfies (20), for the parameter groups of *UP* and* PG* and small *ε* > 0, the system ((10a) and (10b)) has at least 22 limit cycles with the configuration shown in Figure 5.

## 4. The Analysis of Stability

Based on the local and global bifurcation theory and the results of paper [14, 18], we have two propositions which describe the properties of the detection function at the boundary values of *h*.

**Theorem 4 (the parameter value of Hopf bifurcation)**

Suppose that, as *h* → *h*_1_, the periodic orbit Γ^*h*^ of ((11a) and (11b))_*ε*=0_ approaches a singular point (*ξ*, *η*). Then at this point the Hopf bifurcation parameter value is given by

(21) $$\begin{array}{c} b_{H}=\lambda \left(h_{1}\right)+O\left(\varepsilon \right)=\underset{h\to h_{1}}{\lim}\lambda \left(h\right)+O\left(\varepsilon \right)=F\left(\xi ,\eta \right)+O\left(\varepsilon \right). \end{array}$$

**Theorem 5 (bifurcation direction of heteroclinic or homoclinic loop)**

Suppose that, as *h* → *h*_2_, the periodic orbit Γ^*h*^ of ((11a) and (11b))_*ε*=0_ approaches a heteroclinic (or homoclinic) loop connecting a hyperbolic saddle point (*α*, *β*), where the saddle point value satisfies

(22) $$\begin{array}{c} SQ\left(\alpha ,\beta \right)=2\varepsilon \sigma \left(\alpha ,\beta \right)\equiv 2\varepsilon \left(\lambda \left(h_{2}\right)-F\left(\alpha ,\beta \right)\right)>0\left(<0\right). \end{array}$$

Then one has

(23) $$\begin{array}{c} \lambda^{\prime }\left(h_{2}\right)=\underset{h\to h_{2}}{\lim}\lambda^{\prime }\left(h\right)=-\infty \left(+\infty \right). \end{array}$$

From the theorems above, we can also prove the following results.

1. If Γ^*h*^ contracts inwards as *h* increases, then the stability of limit cycles mentioned in Theorem 4 and the sign of *λ*′(*h*_2_) in Theorem 5 have the opposite conclusion.
2. If the curve Γ^*h*^ defined by *H*(*x*, *y*) = *h*  (*h* ∈ (*h*_1_, *h*_2_)) consists of *m* components of oval families having *Z*_*q*_-equivariance, then Theorem 4 gives rise to simultaneous global bifurcations of limit cycles from all these *m* oval families.
3. If ((11a) and (11b))_*ε*_  has several different period annuluses filled with periodic orbit families {Γ_*i*_^*h*^}, then, by calculating detection functions for every oval family, the global information of bifurcations of system ((11a) and (11b))_*ε*_ can be obtained.

On the basis of the method of the theorems above, we have the following analyses of stability.

1. If the period orbit Γ^1^ expands outwards as *h* increases and *λ*_1_(*h*_1_) < 0, then the period orbit Γ^1^ is stable.
2. If the period orbit Γ^2^ expands outwards as *h* increases and *λ*_2_(*h*_2_) > 0, then the period orbit Γ^2^ is unstable.
3. If the period orbit Γ^5^ contracts inwards as *h* increases and *λ*_5_(*h*_4_) > 0, then the period orbit Γ^5^ is stable.
4. If the period orbit Γ^6^ contracts inwards as *h* increases and *λ*_6_(*h*_4_) < 0, then the period orbit Γ^6^ is unstable.
5. If the period orbit Γ^8^ contracts inwards as *h* increases and *λ*_8_(*h*_5_) > 0, then the period orbit Γ^8^ is stable.

**Proposition 6**

**Figure 6:**
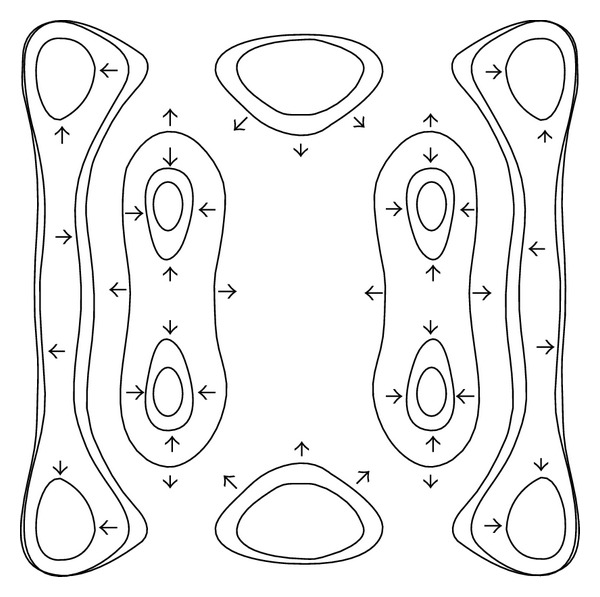
The stability of 22 limit cycles of system ((10a) and (10b)) with parameter conditions* UP* and* PG *is given.

It follows that, under the parameter conditions of* UP* and* PG*, 10 of the 22 limit cycles are stable and others are unstable with the configuration shown in Figure 6.

## 5. The Physical Meaning of Tokamak Equations under the* UP* and* PG* Parameter Groups

The relationship between the actual parameters of the physical equations and the parameter conditions* UP* and* PG* will be discussed in the following parts and the results will be explained following the theory of multiple limit cycle bifurcation.

**Proposition 7**

One has the following parameters relationship between ((10a) and (10b)) and (5):

(24) $$\begin{array}{c} \overset{10}{\underset{i=1}{\sum }}A_{i}^{\left(k\right)}x_{i}+\sum_{\begin{array}{c} 1\leq l1\leq 10\\ 1\leq l2\leq 10 \end{array}}C_{l1l2}^{\left(k\right)}\overset{2}{\underset{i=1}{\prod }}x_{li}=b^{\left(k\right)}\;\left(k=1,2,\ldots ,9\right), \end{array}$$

where

(25) {xi ∣ i=1,…,10}={μ,σ,(δ1+δ2),α2,α3,β2,β3,β4,β5,β6},C(1)=(C5,5(1),C5,8(1),C8,8(1),C9,9(1),C9,10(1),C10,10(1))=(3,2,−11,7,2,−9),b(1)=−60.16,  A5(2)=36,A8(2)=12,  A(2)=(A5(2),A8(2))=(36,12),C(2)=(C1,9(2),C2,8(2),C2,5(2),C3,8(2),C3,5(2),C4,6(2),C4,4(2),C6,6(2),C7,7(2))=(12,12,−36,−3,3,40,40,16,4), b(2)=131.76,C(3)=(C1,9(3),C2,8(3),C2,5(3),C3,5(3),C3,8(3),C4,6(3),C4,4(3),C7,7(3),C6,6(3))=(12,12,−36,3,3,40,40,4,16),A(3)=(A8(3),A5(3))=(12,36),  b(3)=192.24,C(4)=(C5,10(4),C5,9(4),C8,10(4),C8,9(4))=(3,5,13,3),C(5)=(C8,9(5),C8,10(5),C5,9(5),C5,10(5))=(1,1,1,1),b(5)=33.8,C(6)=(C1,5(6),C1,8(6),C2,10(6),C3,7(6),C3,10(6),C4,7(6),C6,7(6))=(12,12,48,1,−15,8,8),A(6)=(A10(6),A9(6))=(24,8),  b(6)=121.25296,C(7)=(C8,9(7),C8,10(7),C5,9(7),C4,10(7))=(1,−9,−1,1),b(7)=23.07.

**Proposition 8**

Based on Proposition 7, (5), and ((4a) and (4b)), one has the following results:

(26) ∑i=112Aikxi+∑1≤l1≤121≤l1≤12Cl1,l2k∏i=12xli+∑1≤l1≤121≤l2≤121≤l3≤12Dl1,l2,l3,l4(k)∏i=13xli  +∑1≤l1≤121≤l2≤121≤l3≤121≤l4≤12El1,l2,l3,l4(k)∏i=14xli=0,

where

(27) {xi ∣ i=1,…,12}={bi,aj ∣ i=0,…4,j=2,…8},C(1)=(C2,2(1))=(−0.9761411),E(1)=(E2,3,3,8(1),E2,2,3,6(1),E1,2,3,10(1),E1,3,3,9(1))=(1,−1,2,−3),C(2)=(C2,2(2))=(−5.124814),D(2)=(D2,3,10(2),D3,3,9(2))=(1,−1),E(2)=(E1,2,4,8(2),E2,2,4,7(2),E1,1,4,9(2))=(2,−1,3),D(3)=(D2,2,3(3),D2,2,7(3),D1,2,8(3),D3,3,9(3))=(1,1,−2,3),C(4)=(C2,8(4),C1,9(4),C2,2(4))=(1,−3,−2.493826),C(5)=(C2,2(5))=(−3.988565),D(5)=(D2,2,4(5),D3,3,9(5))=(3,3),A6=(A9(6))=(−1),  C(6)=(C2,2(6))=(0.4428814),A(7)=(A2(7))=(−2.502493),D(7)=(D1,3,12(7),D2,3,11(7),D3,5,8(7),D2,5,6(7),D1,5,10(7))=(2,−1,2,−1,2).

**Proposition 9**

With the help of Propositions 7 and 8 above, one has the following results:

(28) ∑i=117Ai(k)xi+∑1≤l1≤171≤l1≤17Cl1,l2(k)∏i=12xli+∑1≤l1≤171≤l2≤171≤l3≤17Dl1,l2,l3(k)∏i=13xli+∑1≤l1≤171≤l2≤171≤l3≤171≤l4≤171≤l5≤17El1,l2,l3,l4(k)∏i=14xli+∑1≤l1≤171≤l2≤171≤l3≤171≤l4≤171≤l5≤17Fl1,l2,l3,l4,l5(k)∏i=15xli=0,

where

(29) {xi ∣ i=1,…,17}={D−,D+,G,A,A˙,A¨,V0,V˙0,f˙1,γ,g0,β,G¨,α,μ1,G˙,f1},A(1)=(A8(1),A11(1))=(−3,−1),C(1)=(C3,10(1))=(1.655139),A(2)=(A5(2))=(−3),  C(2)=(C3,10(2))=(2.472232),C(3)=(C3,14(3),C13,15(3),C3,10(3))=(1,1,−0.643029),A(4)=(A10(4),A12(4))=(1.004536,1),E(5)=(E2,4,8,8(5))=(1),F(5)=(F2,3,5,7,8(5),F2,3,6,7,7(5),F2,5,7,7,16(5))=(−4,1,1),D(6)=(D4,7,7(6))=(2.447986),E(6)=(E1,5,5,7(6),E1,4,5,8(6))=(2,−4).

**Proposition 10**

With the help of algebraic method and Lingo mathematical software, one can get that *D*_max⁡_ = 2.516874 and *D*_min⁡_ = 2.4138866 under the parameter conditions of* UP* and* PG*, where *D*_max⁡_ and *D*_min⁡_, respectively, denote the diffusion coefficients of H-mode and L-mode. This shows that, when it satisfies the conditions of *D*_max⁡_ = 2.516874 and *D*_min⁡_ = 2.4138866, there will be 22 limit cycles in the physical model of thermonuclear reaction in Tokamak, 10 of which are stable and the others are unstable. The structure and morphology of limit cycles provide a theoretical basis for the improvement of Tokamak nuclear device.

## 6. Conclusions

This paper focuses on the bifurcations of multiple limit cycles for a Tokamak system. First, the method of multiple scales and normal form theory are employed to obtain the average equation in the Tokamak system, which has the form of a *Z*_2_-symmetric perturbed polynomial Hamiltonian system of degree 5. Then, with the bifurcation theory of planar dynamical system and the method of detection functions, the bifurcations of multiple limit cycles of the averaged equation are analyzed. Finally, the dynamical behavior of the Tokamak system under a group of parameters condition is given.

One control condition of parameters is given to obtain 22 limit cycles of the Tokamak system. Ten of them are stable and the others are unstable. The Hopf bifurcation and limit cycles in averaged equation ((10a) and (10b)) correspond to the L-mode to H-mode transition near the plasma edge in Tokamak. It implies that the amplitude modulated oscillations can jump from one limit cycle to another with a change of the initial conditions. Because different limit cycles are located in different energy planes of ((10a) and (10b)), motion of the Tokamak system can jump from a lower energy plane to a higher energy plane.

## Appendices

### A.

The coefficients given in ((4a) and (4b)) are as follows:

(A.1) D±=12(Dmax⁡±Dmin⁡),  a0=D−A(V¨0−2V˙2V0),a1=D−A[G˙V˙0+G(V¨0−2V0V˙02)],a2=D−A[G˙A˙+GA¨−2GV0(2A˙V˙0−AV0V˙02)],a3=D+A[A¨−2V0(2A˙V˙0−AV0V˙02)],a4=−2D+A˙AV0(A˙−2V0AV˙0),  a5=2D+A˙2V02,a6=−2D−GA˙AV0(A˙−2V0AV˙0),a7=−2f1V0,  a8=−f1A,  a9=−f1AV02,b0=g0+3V˙0γG,  b1=3A˙γG,b2=1γ(α+μ1GG¨),  b3=−βγ,  b4=f2γG.

### B.

The coefficients given in (5) are as follows:

(B.1) ω2=a3b2−a1b1+a2b0−2b1a4b2b0+6b12a5b2b02−a6b1b02,α2=a4b1b22−a2b2+2b1a6b2b0−3b12a5b22b0,α3=2b1a4b3b0−a3b3+a6b1b2−a5b12(b22−3b3b02),μ=b2+a3−2a4b1b0+3b12a5b22,  β2=a4b1−3b12a5b0,β3=a2−2b1a4b2−2a6,  β4=a6b1−3b12a5b0,β5=3b3+3b12a5b22,β6=a5b12,  δ1=2b1a8b2b0−a7b2,δ2=2b1a4b4b2−a2b4+2b1a6b4b0,F1=a9b1−a7b0+a8b1b02,F2=[(2b1a4b4b0−a3b4−3b12a5b4b02)2+(b4Ω2)2]1/2,φ0=arctan(b4Ω2(2/b1)a4b4b0−a3b4−(3/b12)a5b4b0),α0=a0b1−a3b0−a7b0+a4b1b02+a8b1b02−a5b12b03.

### C.

The coefficients given in ((10a) and (10b)) are as follows:

(C.1) a01=−112β3F2sin⁡(φ2)+13β2F2cos⁡⁡(φ2)+16α2F2cos⁡⁡(φ2)−164(δ1+δ2)2+18μ2−σ,a03=12β4+32α3+12β5μ+12β4σ+16β32+23β22+53α2β2−32α3σ+53α22−18β4(δ1+δ2)+18α3(δ1+δ2),a05=916β62−58β5β6+116β52−516β42+18α3β4+316α32−98β62+34β5β6+38β52−38β42,b10=12μ+112β3F1+112β3F2cos⁡(φ2)+13β2F2sin(φ2)+16α2F2sin(φ2),b30=−12β4−32α3−12β5μ−12β4σ−16β32−23β22−53α2β2+32α3σ−53α22−18β4(δ1+δ2)−18α3(δ1+δ2),b50=38β4β5+138β4β6+58α3β5+38α3β6,a50=98β4β5−18β4β6+78α3β5+98α3β6,a41=−2716β62+38β5β6+2116β52−3316β42−158α3β4+6316α32+54α3β3+34α3β6+94β4β5−14β4β6,a32=−92β4β5+12β4β6−72α3β5−92α3β6,a23=−454β62+10β5β6+54β52+54β42−54α3β4+154α32−154α3β4,a14=118α3β5+218α3β6+218β4β5−298β4β6,a30=32β6+12β5+3β6σ+34β4μ+12β2β3+12α2β2+34α3μ−12β6(δ1+δ2),a21=12β4+32α3+12β5μ+12β4σ+16β32+23β22+53α2β2−32α3σ+53α22−58β4(δ1+δ2)−38α3(δ1+δ2)+34α33(δ1+δ2),a12=32β6+12β5+3β6σ+34β4μ+12β2β3+12α2β3+34α3μ+14β5(δ1+δ2)−34β6(δ1+δ2),a10=12μ−112β3F1−112β3F2cos⁡(φ2)−13β2F2sin(φ2)−16α2F2sin(φ2),b14=6316β62−318β5β6−116β52−1916β42+118α3β4−316α32,b23=114α3β5+214α3β6+214β4β5−294β4β6,b32=−278β62+194β5β6−118β52+318β42+14α3β4−338α32,b41=18α3β5−98α3β6−98β4β5+418β4β6,b03=32β6+12β5+3β6σ+34β4μ+12β2β3+12α2β3+34α3μ−14β6(δ1+δ2)−34β6(δ1+δ2),b12=−12β4−32α3−12β5μ−12β4σ−16β32−23β22−53α2β2+32α3σ−53α22+118β4(δ1+δ2)+38α3(δ1+δ2)+34α33(δ1+δ2),b21=32β6+12β5+3β6σ+34β4μ+12β2β3+12α2β3+34α3μ−14β5(δ1+δ2)+94β6(δ1+δ2),b01=12μ+112β3F1+112β3F2cos⁡(φ2)+13β2F2sin(φ2)−16α2F2sin(φ2).
